# Socioeconomic differences in tobacco outlet presence, density, and proximity in four cities in the Netherlands

**DOI:** 10.1186/s12889-023-16347-7

**Published:** 2023-08-09

**Authors:** Tessa R.D. van Deelen, Els M. Veldhuizen, Bas van den Putte, Anton E. Kunst, Mirte A.G. Kuipers

**Affiliations:** 1 Department of Public Health, Amsterdam Public Health research institute, Amsterdam UMC, University of Amsterdam, Postbus, Amsterdam, 22660, 1100 DD the Netherlands; 2 Department of Geography and Planning, University of Amsterdam, Amsterdam, Postbus, Amsterdam, 15629, 1001 NC The Netherlands; 3 Amsterdam School of Communication Research, University of Amsterdam, Amsterdam, Postbus, Amsterdam, 15791, 1001 NG The Netherlands

**Keywords:** Tobacco outlet, Presence, Density, Proximity, Socioeconomic, Inequality

## Abstract

**Background:**

Previous studies found that tobacco outlets were unevenly distributed by area socioeconomic status (SES). However, evidence from continental Europe is limited. This study aims to assess differences in tobacco outlet presence, density and proximity by area SES in the Netherlands.

**Methods:**

All tobacco outlets in four Dutch cities (Amsterdam, and medium-sized cities Eindhoven, Haarlem, and Zwolle) were mapped between September 2019 and June 2020. We estimated associations between average property value of neighbourhoods (as an indicator of SES, grouped into quintiles) and (1) tobacco outlet presence in the neighbourhood (yes/no), (2) density (per km^2^), and (3) proximity to the closest outlet (in meters), using logistic and linear regression models.

**Results:**

46% of neighbourhoods contained at least one tobacco outlet. Tobacco outlets were mostly situated in city centres, but the distribution of tobacco outlets varied per city due to differences in urban structures and functions. In the medium-sized cities, each quintile higher neighbourhood-SES was associated with lower tobacco outlet presence (OR:0.71, 95%CI:0.59;0.85), lower density (B:-1.20 outlets/km^2^, 95%CI:-2.20;-0.20) and less proximity (B:40.2 m, 95%CI 36.58;43.83). Associations were the other way around for Amsterdam (OR:1.22, 95%CI:1.05;1.40, B:3.50, 95%CI:0.81;6.20, and B:-18.45, 95%CI:-20.41;-16.49, respectively). Results were similar for most types of tobacco outlets.

**Conclusion:**

In medium-sized cities in the Netherlands, tobacco outlets were more often located in low-SES neighbourhoods than high-SES. Amsterdam presented a reverse pattern, possibly due to its unique urban structure. We discuss how licensing might contribute to reducing tobacco outlets in low-SES neighbourhoods.

**Supplementary Information:**

The online version contains supplementary material available at 10.1186/s12889-023-16347-7.

## Introduction

Most countries have insufficiently regulated the tobacco retail environment [1]. Tobacco outlets are ubiquitous [2]. This is problematic as exposure to tobacco products is associated with pro-smoking cognitions,[3, 4] and may increase smoking susceptibility,[4] initiation,[5] and smoking prevalence [6]. Moreover, it may frustrate cessation attempts [7, 8]. Similarly, close proximity of tobacco outlets to the home environment is associated with adverse smoking outcomes [9].

Variation in tobacco outlet density and proximity by sociodemographic neighbourhood characteristics may contribute to inequalities in smoking initiation and prevalence between socioeconomic groups [5, 10]. To inform tobacco control policies aimed at the retail environment, it is essential to have accurate knowledge of where tobacco outlets are located to determine whether the impact of these policies will be equitably greater in more disadvantaged areas. However, globally, data are inadequate [11].

Studies in the United States (US),[12–14] Canada,[15] New-Zealand,[16] and Australia [17] found higher tobacco outlet density in more deprived areas. Similar results were found in Scotland, where there were more tobacco displays [18] and larger clusters of tobacco outlets [19] in deprived areas. Compared with children living in the least deprived areas, children from deprived areas in Scotland were seven times more likely to be exposed to tobacco retailers due to significantly larger numbers of outlets in their neighbourhood [20]. In mainland Europe, a study in Cologne Germany also found higher tobacco outlet density in more deprived city districts [21]. Neighbourhood differences in tobacco outlet proximity are unknown.

In Europe, few studies examined spatial patterns of tobacco outlets and resulting socioeconomic patterns. Moreover, these studies focus solely on tobacco outlet density and not on proximity. However, both measures are important as they demonstrate different risks [22]. Density is a measure capturing a degree of tobacco outlet provision in a given area (affecting individuals’ risk of exposure), while proximity is a measure indicating accessibility (affecting individuals’ closeness to tobacco outlets) [23]. Moreover, density measures may be bounded by administratively defined borders, while proximity is not [24]. This is an advantage because administratively defined borders are not an actual boundary to access tobacco outlets. In addition, both measures may be differently impacted by tobacco control policies, dependent on tobacco retailer concentrations within cities [25].

In the Netherlands, there is no registration or licencing system for tobacco outlets and, at the time of this study, no policies were implemented to reduce the number of tobacco outlets. However, the Netherlands aims for a smoke-free generation in 2040. In this regard, a tobacco sales ban in supermarkets is planned for 2024, and government intends to further ban tobacco sales in petrol stations and small outlets after 2030. Therefore, this study may contribute to improving monitoring and evaluation of tobacco control policies aimed at the point of sale, and to eliminating socioeconomic neighbourhood inequalities in tobacco availability [11]. This study aimed to visualise and quantify spatial patterns of both tobacco outlet density and proximity by neighbourhood socioeconomic status in four Dutch cities. In doing so, this study is the first in continental Europe to assess socioeconomic patterns of both tobacco outlet density and proximity.

## Methods

Between September 2019 and June 2020, an observational audit of retail outlets was performed in four Dutch cities, Amsterdam, Eindhoven, Haarlem, and Zwolle. Independent observers identified tobacco retailers by systematically walking through distinct neighbourhoods, covering all streets. In total, 23 observers were employed. All possible tobacco outlets were entered to check for tobacco sales. Stores selling tobacco were identified as tobacco retailers. Their locations were located on a map using Esri’s ArcGIS Collector mobile app version 19.0.2 [26].

After auditing the four cities, we revisited 11% of administratively defined areas (between seven to ten areas per city), locating 26% (n = 225) of all identified tobacco outlets. To check for observer reliability, another observer checked for missed tobacco. In one of the four cities, three new outlets (1.3%) selling tobacco through vending machines were found and added on the map. The percent agreement was 98.7%.

Statistics Netherlands provided datasets with socioeconomic characteristics of the four cities. We used data on neighbourhood level and postal code level (PC6). Neighbourhoods are administratively defined areas, for example based on buildings’ construction year, the type of buildings, and natural borders, and were created for municipalities’ administrative purposes. PC6 is the smallest administratively defined level containing all four numerical digits and both letters of the Dutch postal code system. It corresponds to a street side or block of houses, and was created for mail delivery purposes. PC6 areas are not necessarily nested within neighbourhoods. For both levels, the PC6 and neighbourhoods differ in surface area, but are comparable in terms of population density. The most recent comprehensive publicly available dataset for neighbourhoods was 2020. For PC6 2016.

### Study population

The number of included neighbourhoods and PC6 areas were in Amsterdam 463 and 18,188, in Eindhoven 116 and 5508, in Haarlem 111 and 4056, and in Zwolle 78 and 3344. The number of tobacco outlets in each city was 587, 131, 82, and 70, respectively (Supplementary Table 1).

### Variables

All outlets selling tobacco were categorised into the following types; supermarkets (regular supermarkets and small supermarkets ‘to go’), petrol stations, small shops (convenience stores, newsagents, bookstores, telephone stores, liquor stores, and night shops), hospitality venues (snack bars, bars/cafés/restaurants, casinos, hotels), and tobacco specialist shops (i.e., selling mainly tobacco and related products, and at least 10 square metres).

The socioeconomic status (SES) of each neighbourhood and PC6 area was based on the average property value of the area, in euros, and based on residential properties only, which was the only indicator of SES on neighbourhood level for which sufficient and high quality data was available. SES was divided in quintiles within each city.

Using Geographic Information System (GIS) software ArcMap for Desktop version 10.4.1, the number, presence, density, and proximity of tobacco outlets were calculated. The number of tobacco outlets was measured as the number of outlets within each city, and as a neighbourhood mean for each city. A binary variable presence (yes/no) was created to indicate neighbourhoods with at least 1 tobacco outlet.

Density refers to the provision of tobacco outlets and is often measured as the total number of outlets per square kilometre [23]. Density was calculated for each neighbourhood as the number of tobacco outlets per square kilometre (km^2^). Per city, density was calculated as the mean density across all neighbourhoods with at least 1 tobacco outlet.

Proximity indicates closeness and ease of accessing tobacco [23]. Proximity was calculated as the shortest Euclidean distance in metres from the centre of a PC6 area to the nearest tobacco outlet. Per city, we calculated proximity as the mean of all PC6 areas. For both density and proximity, calculations were repeated for each type of tobacco outlet per city. For Amsterdam, we performed separate calculations without the city centre, because the city centre is a very dissimilar area due to its function as a tourist area.

### Analyses

ArcMap for Desktop 10.4.1 was used to map the distribution of tobacco outlets in each city. Per city, we created a map visualising the distribution of tobacco outlets per SES quintile on PC4 level. This level is larger than the neighbourhood level to maintain readability of the maps.

For each city, the association between the independent variable SES in quintiles and the dependent variable presence (yes/no) was assessed on neighbourhood level with a logistic regression analysis to indicate the odds of containing at least 1 tobacco outlet with each increase in SES quintile. Linear regression analyses were used to assess the association between SES in quintiles and density (/km^2^) for each neighbourhood with at least 1 tobacco outlet, and between SES of the PC6 in quintiles and proximity (i.e., the average distance from the centre of the PC6 to the nearest tobacco outlet). The analyses were performed using SPSS Statistics version 28.

## Results

Table 1 provides for each city the neighbourhood-SES in average property value (x €1,000), and the four tobacco outlet measures. The average property value in medium-sized cities is 334.9 and 451.6 in Amsterdam. On average, there were more tobacco outlets per neighbourhood in Amsterdam (1.27) than in medium-sized cities (0.92). Less than a half of neighbourhoods contained at least one tobacco outlet (36-48%). In those neighbourhoods, the average tobacco outlet density was highest in Amsterdam (18.6/km^2^) and lowest in Eindhoven (5.40/km^2^). The distance to any tobacco outlet was on average 310 m. In Amsterdam, distance was smallest (250 m). In Zwolle, people lived on average further away from tobacco outlets (513 m).

**Table 1:** Neighbourhood characteristics for mean property value and tobacco outlets by city (area)

	Property value (x€1,000) per neighbourhood	Tobacco outlets per neighbourhood	Neighbourhoods with ≥ 1 outlet	Density (/km^2^) per neighbourhood ^a^	Distance (metres) in PC6
**City**	Mean (SD)	Mean (SD)	N (%)	Mean (SD)	Mean (SD)
Medium-sized cities	334.9 (148.9)	0.92 (1.52)	126 (41.3)	6.79 (6.92)	393.9 (315.9)
Eindhoven	313.6 (151.7)	1.13 (1.62)	56 (48.3)	5.40 (6.78)	368.5 (216.6)
Haarlem	372.3 (137.9)	0.71 (1.25)	40 (36.0)	9.53 (6.66)	330.1 (225.7)
Zwolle	312.1 (151.2)	0.90 (1.69)	30 (38.5)	5.72 (6.64)	513.0 (475.3)
Amsterdam	451.6 (227.4)	1.27 (2.03)	224 (48.4)	18.64 (26.36)	250.1 (198.3)
Excl. city centre	431.0 (234.8)	1.05 (1.72)	174 (44.3)	11.72 (10.30)	266.7 (203.8)

Note. PC6 = smallest administratively defined area containing all four numerical digits and both letters of the Dutch postal code system

^a^ Excluding neighbourhoods without tobacco outlets

**Fig. 1:**
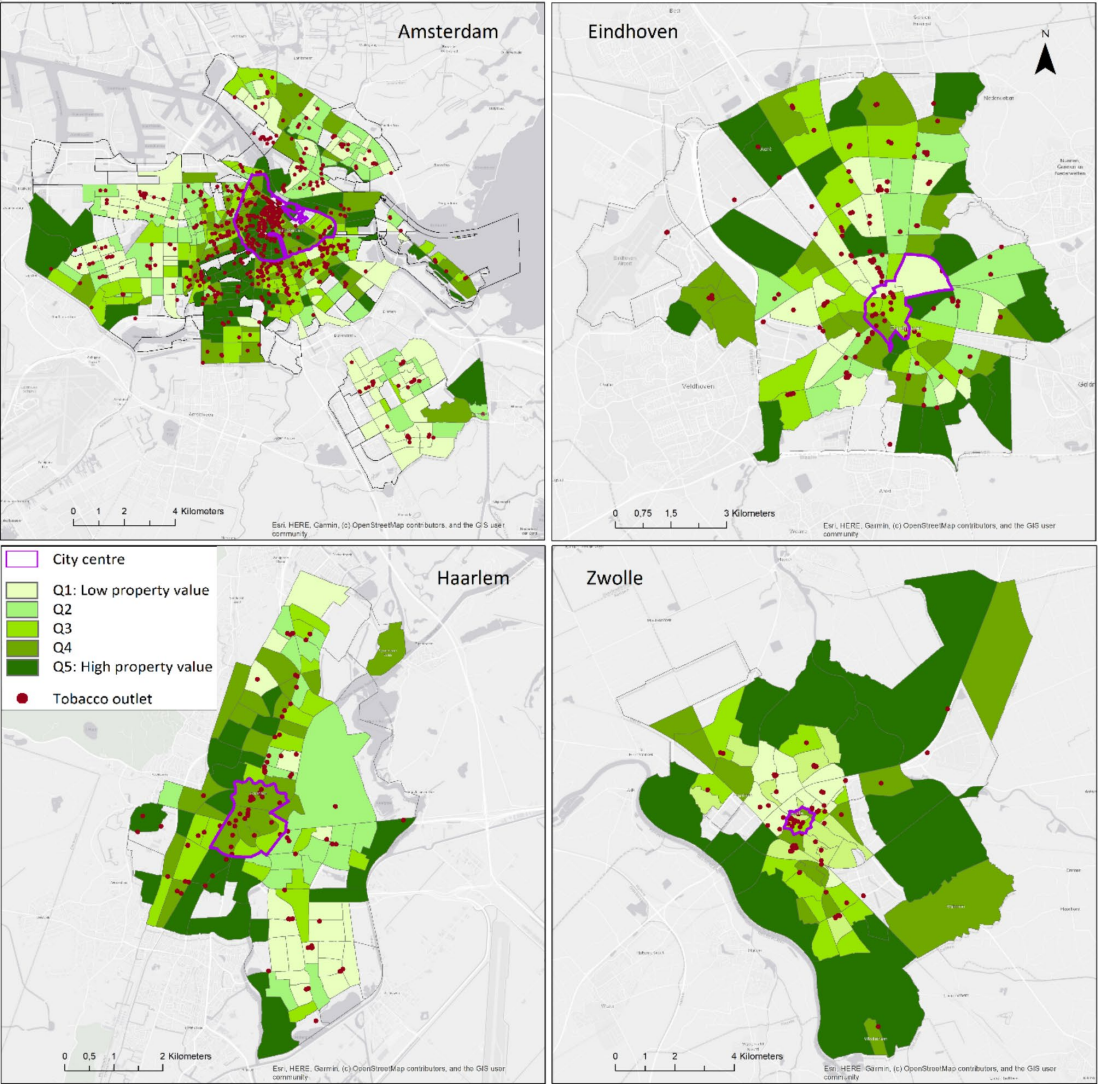
Distribution of tobacco outlets per neighbourhood property level in quintiles

The spatial distribution of tobacco outlets shows different patterns per city (Fig. 1). In Haarlem, tobacco outlets were mainly located along a main road, whereas in Zwolle, they were more clustered in the city centre. Tobacco outlets in Eindhoven clustered in the city centre and in shopping centres in suburban areas. In Amsterdam, there was a very high concentration of tobacco outlets in the city centre, and a high concentration in surrounding areas, while in other areas tobacco outlets clustered near shopping centres and along main roads. The colours indicate the level of SES. In Zwolle, tobacco outlets were mainly concentrated in areas with lower SES, while in Haarlem and Eindhoven, distribution of tobacco outlets was more scattered over areas with different levels. In Amsterdam, tobacco outlets were mainly concentrated in the high-SES city centre.

**Fig. 2:**
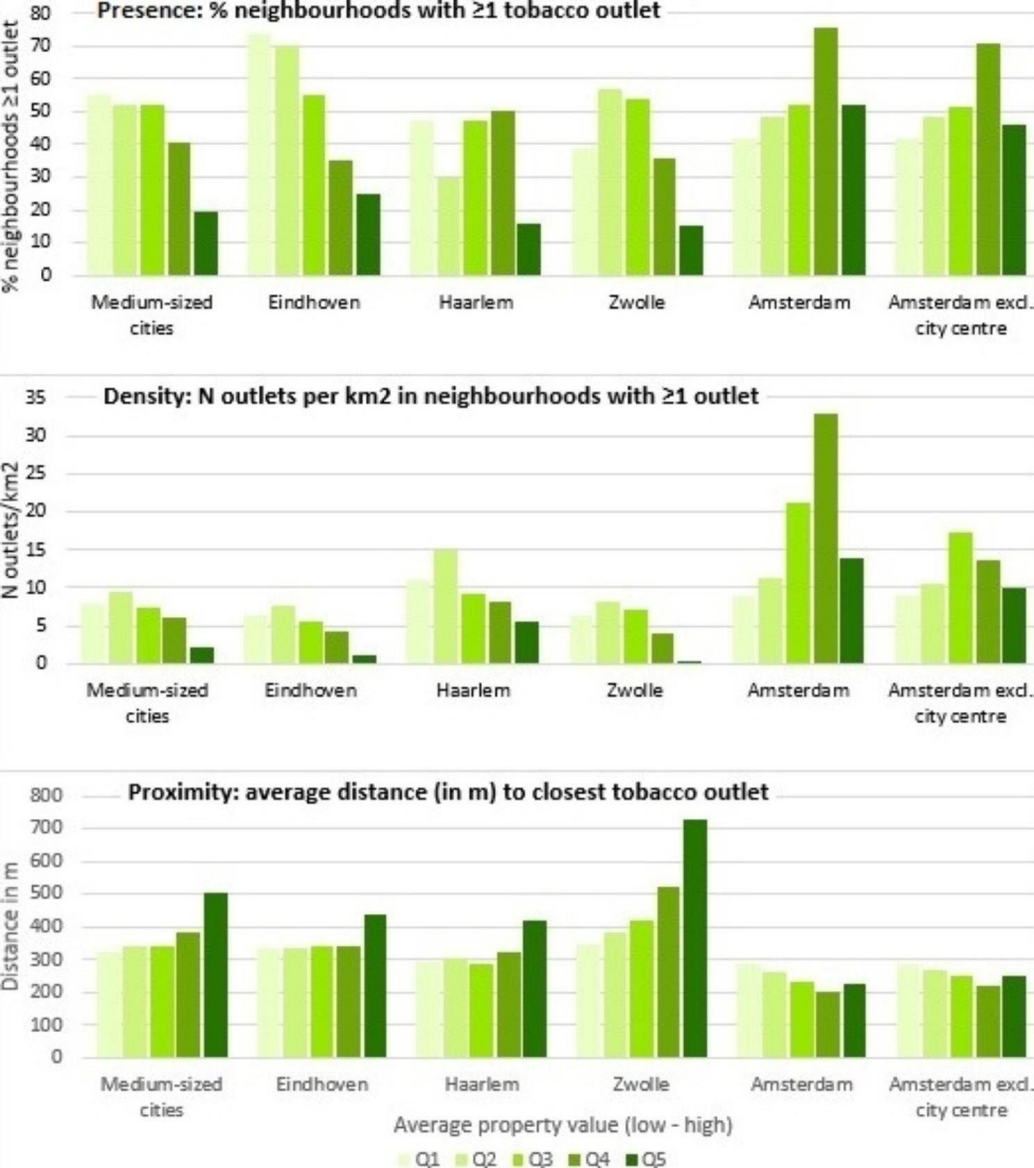
Tobacco outlet presence, density, and proximity, per quintile property value, stratified by area/city

The graphs in Fig. 2 picture the presence (% neighbourhoods with ≥ 1 outlet), density (/km^2^), and distance (in metres) to tobacco outlets per quintile neighbourhood-SES, stratified per area/city. Presence decreased with higher neighbourhood-SES in Eindhoven, but less so in Haarlem and Zwolle, and not in Amsterdam. No patterns were seen for the average density of tobacco outlets in those neighbourhoods. The average shortest distance to tobacco outlets tended to increase with higher SES in the medium-sized cities, but to decrease in Amsterdam.

Table 2’s first column shows the results of the logistic regression analysis for the association between each city’s neighbourhood-SES and tobacco outlet presence (yes/no). For Eindhoven, Haarlem, and Zwolle, with each quintile increase in neighbourhood-SES, the odds of tobacco outlet presence was lower, with respectively 44% (95%CI (Confidence Interval): 0.41;0.77), 16% (95%CI: 0.62;1.12), and 25% (95%CI: 0.52;1.07). In Amsterdam, the odds are 22% higher (95%CI: -20.41;-16.49). The linear regression models for the associations between SES and density/km^2^ and distance (in m) also show different outcomes for the medium-sized cities compared to Amsterdam. With each quintile increase in neighbourhood-SES, the density of tobacco outlets in neighbourhoods with at least one tobacco outlet decreased with respectively 1.20, 1.49, and 1.40 per km^2^, while in Amsterdam the density increased with 3.50 per km^2^. The distance to the nearest tobacco outlet increased with higher SES in Eindhoven (19.8 m), Haarlem (26.7 m), and Zwolle (90.6 m), while in Amsterdam, the distance decreased (-18.5 m).

**Table 2:** Regression analyses for the association between property value (in quintiles) and tobacco outlet presence (logistic), density per km^2^ (linear), and distance in metres (linear) per city (area)

	Presence (yes/no)		Density (/km^2^) ^a^		Distance (metres)	
City	OR	(95%CI)	B	(95%CI)	B	(95%CI)
Medium-sized cities	0.71	(0.59;0.85)	-1.20	(-2.20;-0.20)	40.20	(36.58;43.83)
Eindhoven	0.56	(0.41;0.77)	-1.20	(-2.70;0.30)	19.78	(15.82;23.73)
Haarlem	0.84	(0.62;1.12)	-1.49	(-3.15;0.17)	26.65	(21.76;31.54)
Zwolle	0.75	(0.52;1.07)	-1.40	(-3.65;0.84)	90.56	(80.30;100.83)
Amsterdam	1.22	(1.05;1.40)	3.50	(0.81;6.20)	-18.45	(-20.41;-16.49)
Excl. city centre	1.14	(0.97;1.33)	0.74	(-0.45;1.94)	-11.85	(-14.05;-9.66)

Note. Property value calculated for each PC6 area specifically

^a^ Includes neighbourhoods with ≥ 1 tobacco outlet

In Table 3, the analyses are stratified per type of outlet. In medium-sized cities, the odds of presence of a supermarket, small outlet, and hospitality venue decreased between 26 and 33% with increased neighbourhood-SES. In Amsterdam, the odds increased with 37% for tobacco specialist shops. The density of supermarkets and small outlets decreased with higher neighbourhood-SES in medium-sized cities (-0.65 and − 0.44 outlets per km^2^, respectively), while in Amsterdam, the density of tobacco specialist shops and small outlets increased (0.61 and 2.06 outlets per km^2^, respectively). With higher SES, the average shortest distance to each type of outlet increased in medium-sized cities (between 50 and 131 m), while this decreased in Amsterdam for most outlets (between − 19 m and − 421 m). Most associations for petrol stations were non-significant, except for distance in medium-sized cities (50.28). In Supplementary Tables 2, the average number, density, and distance to tobacco outlets is stratified per type of outlet. The mean density of tobacco outlets was highest for small outlets in Amsterdam (9.3/km^2^), and for supermarkets in the medium-sized cities (2.7/km^2^). For both Amsterdam and the medium-sized cities, the average shortest distance was smallest to supermarkets (364 m; 494 m) and largest to tobacco specialist shops (1293 m; 1600 m). The difference in distance was largest for small outlets (372 m; 824 m).

**Table 3:** Regression analyses for the association between property value (in quintiles) and tobacco outlet presence (yes/no) (logistic), density per km^2^ (linear), and distance in metres (linear) per type of tobacco outlet

						Type of tobacco outlet								
City	Supermarkets			Petrol stations			Small outlets		Hospitality venues			Tobacco specialist shops		
Presence (yes/no)	OR	(95%CI)		OR	(95%CI)		OR	(95%CI)	OR		(95%CI)	OR		(95%CI)
Medium-sized cities ^a^	0.67	(0.55;0.83)		1.02	(0.77;1.33)		0.67	(0.51;0.90)	0.74		(0.59;0.94)	0.65		(0.41;1.03)
Amsterdam	1.07	(0.92;1.24)		1.13	(0.80;1.60)		1.04	(0.89;1.21)	1.16		(0.96;1.41)	1.37		(1.07;1.75)
**Density (/km** ^**2**^ **)** ^b^	**B**	**(95%CI)**		**B**	**(95%CI)**		**B**	**(95%CI)**	**B**		**(95%CI)**	**B**		**(95%CI)**
Medium-sized cities ^a^	-0.65	(-1.21;-0.09)		0.06	(-0.13;0.25)		-0.44	(-0.87;-0.00)	-0.08		(-0.65;0.49)	-0.09		(-0.29;0.10)
Amsterdam	0.36	(-0.27;0.99)		0.04	(-0.14;0.22)		2.06	(0.05;4.07)	0.44		(-0.05;0.93)	0.61		(0.00;1.22)
**Distance (m)**	**B**	**(95%CI)**		**B**	**(95%CI)**		**B**	**(95%CI)**	**B**		**(95%CI)**	**B**		**(95%CI)**
Medium-sized cities ^a^	53.25	(48.64;57.86)		50.28	(42.38;58.19)		90.46	(83.49;97.43)	84.70		(75.60;93.80)	131.36		(116.90;145.81)
Amsterdam	-26.87	(-29.41;-24.34)		-24.08	(-30.49;17.68)		-18.89	(-21.74;-16.05)	-71.37		(-76.84;65.90)	-421.22		(-436.11;-406.33)

Note. Property value calculated for each PC6 area specifically

^a^ Medium-sized cities includes Eindhoven, Haarlem, and Zwolle

^b^ Includes only neighbourhoods with a tobacco outlet

## Discussion

### Key results

In this study, we described spatial patterns of tobacco outlets in four Dutch cities, and we assessed socioeconomic differences in the presence, density, and proximity of tobacco outlets. Tobacco outlets tended to concentrate in city centres, along main roads, and in suburban shopping areas. In the medium-sized cities Eindhoven, Haarlem, and Zwolle, neighbourhoods with lower SES more often contained a tobacco outlet and had higher densities of outlets per km^2^. In Amsterdam, the associations were contrariwise. Similarly, the shortest distance from a postcode to a tobacco outlet decreased with lower SES in medium-sized cities, while it increased in Amsterdam. We found similar results for each type of outlet, except for petrol stations for which no association was found.

### Evaluation of study limitations

As the tobacco retailer audit was done by multiple observers, there is a risk of inter-observer bias. However, as explained in our previous work,[2] objectivity of the observations was high because each observer was trained to use a standardised checklist prior to their work, and only a few discrepancies were found after checking a sample of areas.

The shortest distance to tobacco outlets was measured as Euclidean distance, without taking road networks into account. Therefore, this method does not accurately measure the shortest travel distance to tobacco outlets. However, in Dutch cities, road distance and linear distance are highly correlated, because of dense road networks [27]. Previous studies abroad also found negligible differences between the outcomes of the two methods [28, 29].

We used a single indicator, average property value, to determine neighbourhood socioeconomic status (SES). Housing is one indicator of neighbourhood-SES, and others include income or educational attainment [30, 31]. Combining multiple indicators of SES might give a more nuanced picture that depends less on characteristics of the housing stock [30]. However, property value is proven to be a strong proxy for neighbourhood deprivation [32].

This study was limited to large urban areas in the Netherlands, which may impede generalisability of results to Dutch rural areas or the entire country. Dependent on the built environment and distribution of SES within the municipalities, it is possible to find either a more or less pronounced association between tobacco outlets and neighbourhood SES. Our pilot-observations in seven Dutch rural municipalities showed tobacco outlets in those areas were mainly concentrated in village centres. However, the association with neighbourhood-SES is unknown for rural areas.

### Interpretation

Our results for medium-sized cities are in line with similar studies in Europe. In Cologne, Germany, tobacco outlet presence was higher in less affluent neighbourhoods [21]. Two Scottish studies, in Glasgow and the whole of Scotland, also found higher tobacco outlet density in more deprived urban neighbourhoods [18, 19]. Similarly to our medium-sized cities, in these studies, tobacco outlets were highly concentrated in the city centre and the adjacent residential areas with low-SES, while outlet densities were decreasing towards suburban areas [18, 19, 21]. Also in the United States, tobacco outlet presence and density were higher in low-SES areas, often relatively close to the city centre, with more people living below the poverty line [13] or with low income [14]. Canada,[15] Australia,[17] and New Zealand [16] showed similar patterns.

In the three medium-sized cities, we found that low area SES is associated with higher densities and smaller distances. However, proximity and area SES are stronger associated in Zwolle than in the other two medium-sized cities. Possible explanations may be that, in contrast to Haarlem and Eindhoven, Zwolle is a smaller city, serving a more rural region, in which the fewer service functions are mostly concentrated in the city centre. In Zwolle, large high-SES neighbourhoods are located at the border of the city, far from the centre where most shops are located. Nonetheless, overall results in medium-sized cities are consistent with the finding that, across areas, the distance to tobacco outlets is inversely associated with tobacco outlet density [25]. Unfortunately, no previous study has reported on tobacco outlet proximity in relation to area-level SES. It is however informative to note the correspondence to other types of unhealthy outlets. Fast food outlets were found to be closer to lower-SES neighbourhoods in Canada,[33] the US,[34] and Spain [35].

Contrary to medium-sized cities, we found positive associations in Amsterdam between neighbourhood-SES and tobacco outlet presence, density, and proximity. Residential segregation patterns may explain the particular situation of Amsterdam [36, 37]. Property values are much higher in the city centre and its surrounding than in suburbs [38]. This pattern reflects gentrification processes and policies aimed to diminish area deprivation and to attract new capital to Amsterdam’s centre [39]. As a result, central Amsterdam and its surroundings has attracted high-income residents, while low-income residents had to find new housing farther away. Simultaneously, the city centre of Amsterdam has developed a unique function as tourist area, locating numerous small outlets, such as tourist shops, many of which sell tobacco.

Two mechanisms may explain why tobacco outlet presence, density, and proximity is generally greater in low-SES neighbourhoods. On the one hand, tobacco outlets may purposefully be located in these neighbourhoods, in response to the higher smoking prevalence of the resident population. On the other hand, residents with low-SES may move to areas with high concentrations of tobacco outlets, such as shopping areas, when the housing stock of these areas consist of small houses or apartments with lower property values. However, the latter mechanism depends on the structure and functions of a city at large. The Amsterdam case illustrates that different patterns of residential segregation and outlet distribution may reverse the commonly observed SES differences in outlet presence.

Petrol stations are the exception to the rule that presence, density and proximity are generally higher in low-SES areas. This type of outlet is more often located along main roads in suburbs than in city centres, and thus closer to high-SES residents that tend to live in the suburbs of medium-sized cities.

### Implications

The patterns in medium-sized cities are a concern to public health, because greater exposure to tobacco outlets is associated with smoking behaviour [9, 22]. Residents in low-SES neighbourhoods may have a higher chance to encounter tobacco outlets, increasing their risk of smoking initiation [5] and decreasing the likelihood of smoking cessation [7]. Therefore, SES differences in tobacco outlet presence, density, and proximity may contribute to socioeconomic inequalities in smoking prevalence in adolescents and adults.

Due to this variation, upcoming and intended tobacco sales bans for supermarkets, petrol stations, and small outlets are not expected to address neighbourhood inequalities, as differences in the number of tobacco outlets between low and high-SES neighbourhoods are likely maintained. To achieve a significant absolute reduction of outlets in low-SES neighbourhoods, tobacco control policies should aim to minimise the total number of tobacco outlets per neighbourhood. This could be guaranteed with the introduction of a licensing system with restricted criteria to purchase a license. Without a licensing system, an increase in the number of tobacco specialist shops – exempted from tobacco sales bans – in all neighbourhoods, is very plausible [40]. Therefore, it would help to only allow a maximum number of tobacco outlets per neighbourhood to purchase licenses, only allow specific types of outlets to purchase a license, for instance tobacco specialist shops, and include criteria capping the minimum distance between licensed outlets. The latter restriction would reduce tobacco outlet density especially in low-SES neighbourhoods with high density [40].

## Conclusion

In three medium-sized cities in the Netherlands, tobacco outlets are mainly located in low-SES neighbourhoods, while this was the other way around in Amsterdam. Tobacco licensing with criteria for the total number and density of tobacco outlets per neighbourhood may contribute to equitably reducing tobacco outlets within cities.

### Electronic supplementary material

Below is the link to the electronic supplementary material.

**Supplementary Tables: Supplementary Table 1**. Number of neighbourhoods, postcode areas (PC6), and tobacco outlets per city. **Supplementary Table 2**. Average availability in number, density per km^2^, and distance in metres tobacco outlets per type of outlet per city (area).

## Data Availability

The datasets used and/or analysed during the current study are available from the corresponding author on reasonable request.
