# Supplementary material for: Socioeconomic differences in tobacco outlet presence, density, and proximity in four cities in the Netherlands
Source: BMC Public Health. 2023 Aug 9;23:1515. doi: 10.1186/s12889-023-16347-7 (PMC10413623; doi:10.1186/s12889-023-16347-7)
Supplement: Supplementary file 1 — Supplementary Tables: Supplementary Table 1. Number of neighbourhoods, postcode areas (PC6), and tobacco outlets per city. Supplementary Table 2. Average availability in number, density per km2, and distance in metres tobacco outlets per type of outlet per city (area). [file 12889_2023_16347_MOESM1_ESM.docx]

**SUPPLEMENTARY TABLES**

Supplementary Table 1. Number of neighbourhoods, postcode areas (PC6), and tobacco outlets per city.

|  | **Neighbourhoods** | | |  | **PC6** | |  |  |
| --- | --- | --- | --- | --- | --- | --- | --- | --- |
|  | **Number** | **Area size km^2^** | **Residents** |  | **Number** | **Area size km^2^** |  | **Tobacco outlets** |
| **City** | (N) | mean(SD) | ∑ (mean(SD)) |  | (N) | mean(SD) |  | (N) |
| Medium-sized cities | 305 | 0.79(1.42) | 525605 (1723.3(1398.4)) |  | 12908 | 0.0214 (0.135) |  | 283 |
| Eindhoven | 116 | 0.77(0.74) | 234155 (2018.6(1611.6)) |  | 5508 | 0.022 (0.153) |  | 131 |
| Haarlem | 111 | 0.29(0.32) | 162675 (1465.54(871.34)) |  | 4056 | 0.01  (0.057) |  | 82 |
| Zwolle | 78 | 1.53(2.46) | 128775 (1651.0(1588.9)) |  | 3344 | 0.038 (0.173) |  | 70 |
|  |  |  |  |  |  |  |  |  |
| Amsterdam | 463 | 0.31(0.34) | 869000 (1876.9(1480.3)) |  | 18188 | 0.013  (0.084) |  | 587 |
| Excl. city centre | 393 | 0.34(0.36) | 781810 (1989.3(1533.4)) |  | 15955 | 0.0142  (0.09) |  | 412 |

^*^ Total includes Amsterdam, Eindhoven, Haarlem, and Zwolle.

Supplementary Table 2. Average availability in number, density per km^2^, and distance in metres tobacco outlets per type of outlet per city (area).

|  | **Type of tobacco outlet** | | | | |
| --- | --- | --- | --- | --- | --- |
|  | **Supermarkets** | **Petrol stations** | **Small outlets** | **Hospitality** | **Tobacco specialist shops** |
| **City** | N | N | N | N | N |
| **Tobacco outlets (N)** | 271 | 62 | 303 | 173 | 61 |
| Medium-sized cities ^a^ | 111 | 37 | 51 | 72 | 12 |
| Amsterdam | 160 | 25 | 252 | 101 | 49 |
|  | Mean (SD) | Mean (SD) | Mean (SD) | Mean (SD) | Mean (SD) |
| **Presence (N)** |  |  |  |  |  |
| Medium-sized cities ^a^ | 0.36 (0.68) | 0.12 (0.33) | 0.17 (0.64) | 0.23 (0.64) | 0.04 (0.20) |
| Amsterdam | 0.35 (0.65) | 0.05 (0.23) | 0.54 (1.23) | 0.22 (0.70) | 0.11 (0.38) |
| **Density ^b^ (/km^2^)** |  |  |  |  |  |
| Medium-sized cities ^a^ | 2.7 (3.8) | 0.6 (1.3) | 1.3 (2.9) | 1.9 (3.8) | 0.3 (1.3) |
| Amsterdam total | 4.5 (6.1) | 0.4 (1.8) | 9.3 (19.6) | 2.4 (4.8) | 2.0 (5.9) |
| **Distance (metres)** |  |  |  |  |  |
| Medium-sized cities ^a^ | 494.0 (438.5) | 946.1 (627.3) | 824.4 (626.0) | 867.7 (736.6) | 1599.6 (1147.3) |
| Amsterdam | 363.9 (269.1) | 1044.3 (601.2) | 371.9 (297.3) | 669.4 (530.2) | 1293.2 (1500.1) |

^a^ Medium-sized cities includes Eindhoven, Haarlem, and Zwolle.

^b^ Includes only neighbourhoods with a tobacco outlet.
